# Design, Synthesis and Cytotoxic Evaluation of *o*-Carboxamido Stilbene Analogues

**DOI:** 10.3390/ijms141223369

**Published:** 2013-11-27

**Authors:** Mohamad Nurul Azmi, Mohd Fadzli Md Din, Chin Hui Kee, Munirah Suhaimi, Ang Kheng Ping, Kartini Ahmad, Mohd Azlan Nafiah, Noel F. Thomas, Khalit Mohamad, Leong Kok Hoong, Khalijah Awang

**Affiliations:** 1 Department of Chemistry, Faculty of Science, University of Malaya, Kuala Lumpur 50603, Malaysia; E-Mails: Mohamad.azmi@siswa.um.edu.my (M.N.A.); fadzli_md@yahoo.com (M.F.D.); chkee@um.edu.my (C.H.K.); munirah_oni@siswa.um.edu.my (M.S.); akping_1@hotmail.com (A.K.P.); noelfthomas@um.edu.my (N.F.T.); 2 Department of Chemistry, Faculty of Science and Mathematics, Sultan Azlan Shah Campus, University Pendidikan Sultan Idris, Proton City 35950, Perak Darul Ridzuan, Malaysia; E-Mails: kartini@fmst.upsi.edu.my (K.A.); azlan@fmst.upsi.edu.my (M.A.N.); 3 Department of Pharmacy, Faculty of Medicine, University of Malaya, 50603 Kuala Lumpur 50603, Malaysia; E-Mails: khalitmohamad@um.edu.my (K.M.); leongkh@um.edu.my (L.K.H.)

**Keywords:** *o*-carboxamido stilbenes, amido stilbenes, Heck protocol, cytotoxic effects

## Abstract

Resveratrol, a natural stilbene found in grapes and wines exhibits a wide range of pharmacological properties. Resveratrol is also known as a good chemopreventive agent for inhibiting carcinogenesis processes that target kinases, cyclooxygenases, ribonucleotide reductase and DNA polymerases. A total of 19 analogues with an amide moiety were synthesized and the cytotoxic effects of the analogues on a series of human cancer cell lines are reported. Three compounds **6d**, **6i** and **6n** showed potent cytotoxicity against prostate cancer DU-145 (IC_50_ = 16.68 μM), colon cancer HT-29 (IC_50_ = 7.51 μM) and breast cancer MCF-7 (IC_50_ = 21.24 μM), respectively, which are comparable with vinblastine. The resveratrol analogues were synthesized using the Heck method.

## 1. Introduction

Resveratrol, a popular stilbenoid-type compound exerts a wide range of biological activities, such as anti-carcinogenic, antioxidant, anti-inflammatory, anti-tumor [1,2]. Resveratrol is widely found in grapes and red wine [3,4], and the amount of resveratrol in the skin of fresh grape is between 50–100 mg/g [5]. Resveratrol, a resorcinol derivative was first isolated from the Japanese plant *Veratrum grandiflorum* [6] and was reported as a phytoalexin produced by plants in response to pathogen attack or environmental pressure such as injuries [7]. Resveratrol is also known as a calorie restriction mimetic which is a hypothetical class of drugs that would mimic the substantial anti-aging effects caloric restrictions [8]. Resveratrol is commonly connected to the French Paradox *i.e.*, wine-drinking population with high intake of saturated fat but with few heart related problems, which is attributed to the inhibition of LDL oxidation in human [9]. In relation to cardiovascular disease, an animal model showed that resveratrol reduces blood pressure and cardiac hypertrophy in hypertensive animals as well as protects the heart from ischemia-reperfusion injury and slows the progression of atherosclerosis [10].

The interesting and intriguing biological properties of resveratrol have attracted much attention from synthetic chemists which led to the synthesis of numerous analogues containing diverse range of functional groups. For instance, pterostilbene which is a simple analogue with two methoxyl groups on the aromatic ring have been shown to exhibit good cytotoxicity against estrogen-insensitive breast cancer (MDA-MB-231) [11]. Some naphthyl combretastatin analogues have been reported to inhibit tubulin polymerization [12]. Similar to the naphthalene moiety, stilbene coumarin analogues showed potent antiproliferative activity toward human lung carcinoma cell line (H460) [2]. By substituting the hydroxyl group in resveratrol with an aliphatic acid, positive result was seen in inhibiting cell apoptosis through Toll-like receptor 2 (TLR2) [13]. Another type of analogue, the benzenesulfonamides having a sulfonamide moiety, were tested against 60 human tumor cell lines and were notably cytotoxic toward BT-549 breast cancer and HT-29 colon cancer [14].

**Scheme 1.**
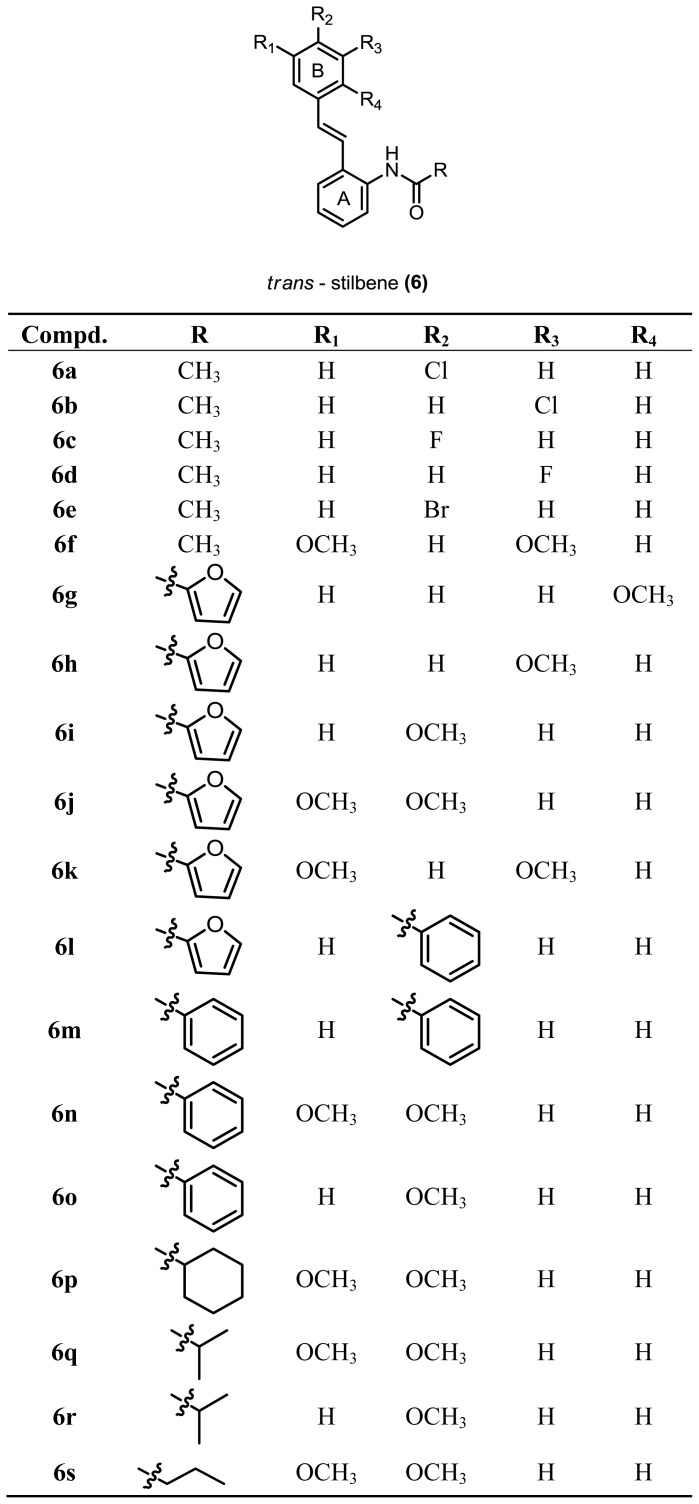
Chemical structures of *o*-carboxamido stilbene analogues mentioned in this study and general structure of the synthesized compounds (**6a**–**6s**).

Previously our group has synthesized a nine *o*-carboxamido stilbene, incorporating the amide moiety and exhibiting good biological activity against a colon cell line (HT-29), liver cell line (HEP-G2), Jurkat cell and murine leukemic cell line [15–17]. In continuation of this study, we have further synthesized more analogues and all these compounds were evaluated for their cytotoxicity against 6 human cell lines; colon cancer (HT-29), estrogen-sensitive breast cancer (MCF-7), estrogen-insensitive breast cancer (MDA-MB-231), prostate cancer (DU-145), pancreatic cancer (BxPC-3) and normal pancreatic cells (hTERT-HPNE). A total of 19 *o*-carboxamido stilbenes were synthesized (**6a**–**6s**) containing different substituted groups linked to the amide group at ring A (Scheme 1). While at ring B, electron donating groups such as methoxy and electron withdrawing group (Cl, Br and F) were attached at different positions.

## 2. Results and Discussion

### 2.1. Synthesis of *o*-Carboxamido Stilbene

**Scheme 2.**
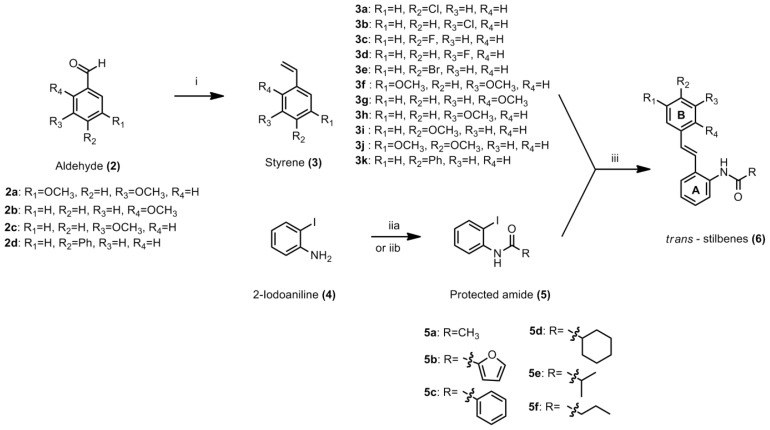
Synthesis of substituted styrenes by Wittig olefination, *o*-carboxamido stilbenes by Heck coupling reaction. Reagents and conditions. (i) H_3_CP(C_6_H_5_)_3_Br, *tert*-BuOK, THF, 24 h, −75 °C; (iia) NaH, acetic anhydride, DMF or (iib) triethylamine (Et_3_N) (0–5 °C), THF, acyl chloride; (iii) triethylamine (Et_3_N), Pd(II) acetate, 120 °C.

The synthesis of *o*-carboxamido stilbenes have been described in Scheme 2. Substituted benzaldehydes **2a**–**2d** were reacted with methyltriphenylphosphoniumiodide in the presence of *tert*-BuOK in a Wittig reaction [16] to provide the styrene derivatives **3f**–**3h** and **3k**; styrene **3a**–**3e**, **3i** and **3j** were purchased from Sigma-Aldrich (St. Louis, MO, USA) and were used without further purification. Iodo carboxamido was prepared in two ways (a) 2-iodoaniline was reacted with NaH and then acetic anhydride was added to give 2-iodoacetamide **5a**; (b) A cooled solution (0–5 °C) of 2-iodoaniline and triethylamine, was added with acyl chloride to give **5b**–**5f**[18]. The iodo carboxamido **5a**–**5f** were coupled with the styrenes **3a**–**3k** using the Heck method by palladium (II) acetate catalyst to provide the (*E*) *o*-carboxamido stilbenes **6a**–**6s** in good yield [16,17].

### 2.2. Cytotoxic Evaluation of *o*-Carboxamido Stilbenes on Selected Human Cancer Cell Lines

**Figure 1.**
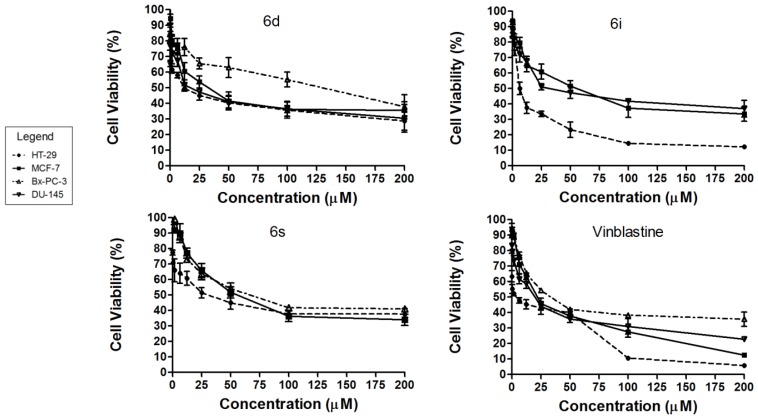

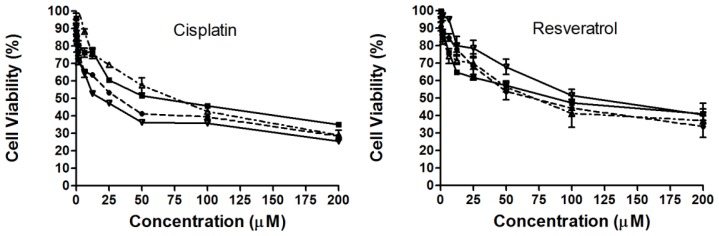
Dose-response curves of selected *o*-carboxamido stilbenes on human cancer cell lines.

Resveratrol, a stilbene with antioxidant and antiproliferative properties, has been shown to inhibit a variety of primary tumors. However, the inhibitory effect is observed only at higher concentration [19,20]. In our effort to improve the cytotoxicity of resveratrol, a total of 19 resveratrol analogues bearing the *o*-carboxamido moiety were synthesized. These analogues together with 3 standards (cisplatin, resveratrol and vinblastine) were screened against 5 human cancer cell lines and one normal human cell line. Of the 19 analogues, 7 (**6d**, **6e**, **6g**–**6i**, **6k** and **6s**) were found to be cytotoxic toward colon cancer cell lines (HT-29), 7 (**6a**, **6d**, **6i**, **6l**, **6n**, **6p** and **6s**) toward estrogen-sensitive breast cancer (MCF-7), none towards estrogen-insensitive breast cancer (MDA-MB-231), 6 (**6d**, **6i**–**6l** and **6p**) toward prostate cancer (DU-145), 2 (**6d** and **6s**) toward pancreatic cancer (BxPC-3) and none towards normal immortalized pancreatic cells (hTERT-HPNE). Analogue **6d** showed cytotoxicity towards almost all cancer cell lines tested except for MDA-MB-231 (Table 1). Dose-response curves of the selected cytotoxic analogues toward HT-29, MCF-7, Bx-PC-3 and DU-145 are shown in Figure 1.

The resveratrol analogue **6n**, which has a bulky phenyl group attached to the amide and two methoxy group at ring B, proved the most potent against estrogen-sensitive breast cancer cell line (MCF-7). The analogue has an IC_50_ of 21.24 μM which is four times lower than that of resveratrol (IC_50_ = 85.71 μM) and comparable with that of vinblastine (IC_50_ = 21.00 μM). Interestingly, all the analogues that possess cytotoxic activity towards MCF-7 lost their inhibition when tested on estrogen-insensitive breast cancer (MDA-MB-231). The differences between these cancer cell lines are that MCF-7 possesses the estrogen receptor whereas MDA-MB-231 does not. This selective cytotoxic activity is also observed with resveratrol where its IC_50_ is 85.71 μM in MCF-7, and 143.57 μM in MDA-MB-231. This observation is due to the similar structure of resveratrol to the synthetic estrogen (diethylstilbestrol) and can act as a weak competitor in binding to the estrogen receptors [21]. The selective cytotoxicity towards estrogen-sensitive breast cancer cell line (MCF-7) lead us to hypothesize that the analogues (**6a**, **6d**, **6i**, **6l**, **6n**, **6p** and **6s**) may have similar estrogen receptor binding properties in MCF-7. Therefore, these analogues may be potential leads for the development of therapeutic agents for hormone-dependent tumors.

The structural activity relationship showed that analogues with a furan moiety at ring A and methoxy group at ring B have improved cytotoxicity toward the colon cancer cell line (HT-29) when compared with resveratrol. Almost all analogues with the furan moiety showed potent cytotoxicity, in particular analogue **6i** (IC_50_ = 7.51 μM) showed ten-fold improved cytotoxicity compared with resveratrol (IC_50_ = 72.9 μM) and four-fold to cisplatin (IC_50_ = 30.61 μM). Significant cytotoxicity is also observed in the prostate cancer cell line (DU-145) with analogue **6d** having an IC_50_ value of 16.68 μM, which represents a six-fold increase in potency compared with resveratrol (IC_50_ = 107.92 μM). This improvement can be attributed to the acetyl group attached to the amide and fluorine, which is an electron withdrawing group at the meta position of the ring B in compound **6d**. Together with **6d** (IC_50_ = 129.78 μM), compound **6s** (IC_50_ = 66.30 μM) having a propyl group attached to the amide, showed selective cytotoxic activity towards the pancreatic cancer cell line, BxPC-3, while sparing (IC_50_ > 200 μM) a normal pancreatic cell line (hTERT-HPNE). All the standards (cisplatin, vinblastine and resveratrol) showed no discrimination in their cytotoxicity between normal and cancer pancreatic cells. In chemotherapy, anticancer drugs that are selective towards cancer cells could reduce severe side-effects toward normal cells in the patient. Therefore, the analogues may offer an improved chemotherapy outcome for cancer patients compared to existing anticancer drugs.

## 3. Experimental Section

### 3.1. General

All spectral data were obtained on the following instruments: Infrared spectra were recorded on a Perkin Elmer FTIR Spectrum RX-1 spectrometer at wavenumber from 4000–400 cm^−1^. Nuclear magnetic resonance (NMR) spectra were obtained on a JEOL JNM-LA 400 and JEOL ECA-400. Spectra are reported in units of ppm on the δ scale, relative to chloroform and the coupling constants are given in Hz. Ultra Violet (UV) spectra were recorded from wavelength 190–400 nm, in methanol, on a Shimadzu UV-Visible Spectrophotometer 1650. Mass spectra were measured using *Agilent* 6530 Accurate-Mass *Q-TOF LC*/*MS* system. Melting points were determined with Mel-Temp II melting point apparatus.

### 3.2. Material

Human cancer cell lines were obtained from American Type Culture Collection (ATCC) (Manassas, VA, USA). Dulbecco’s modified Eagle’s medium (DMEM), 100 mM non-essential amino acids, phosphate buffer solution (pH 7.2), 50 μg/mL gentamicin and 2.5 μg/mL amphotericin B were purchased from Invitrogen Corporation (Carlsbad, CA, USA). 200 mM l-glutamine, foetal bovine serum, 0.25% trypsin-EDTA, dimethyl sulphoxide (DMSO), cisplatin, resveratrol and vinblastine sulphate were purchased from Sigma-Aldrich (St. Louis, MO, USA). MTS [3-(4,5-dimethylthiazol-2-yl)-5-(3-carboxymethoxyphenyl)-2-(4-sulphophenyl)-2*H*-tetrazolium, inner salt] assay kit (CellTiter 96^®^ AQ_ueous_ One Solution) was obtained from Promega (Madison, WI, USA).

### 3.3. Chemical

Unless otherwise noted, materials were purchased from commercial suppliers and used without purification. THF was freshly distilled over calcium hydride. DMF was dried over molecular sieves 4Å (Sigma-Aldrich, St. Louis, MO, USA) prior to use. Column chromatography was performed using Merck silica gel (0.040–0.063 mm). For thin layer chromatography, Merck TLC aluminum sheets (silica gel 60 *F*_254_) were used; centrifugal chromatography, Merck silica gel 60 *PF*_254_ containing gypsum were used.

### 3.4. Procedure for the Preparation of Compounds

#### 3.4.1. General Procedure for Styrene

To a suspension of methyltriphenylphosphonium iodide (1 equiv) in dry THF (25 mL), potassium tert-butoxide (1 equiv) was added in one portion. The mixture was stirred for 1 h at −70 °C to −80 °C. The appropriate aldehyde (1 equiv) was added to the solution. The ice bath was removed and the mixture was allowed to warm to room temperature. After consumption of starting material to form the product, the reaction was quenched with saturated ammonium chloride solution. The mixture was extracted with ethyl acetate and washed with plenty of distilled water. The resulting organic fractions were combined and solvent was removed under reduced pressure to yield crude product. Purification by column chromatography afforded the desired product. The procedure for compounds **3f**, **3g**, **3h** and **3k** is attached in Appendix.

1,3-methoxy-5-vinylbenzene, **3f**. 1H NMR (CDCl_3_, 400 MHz) δ: 6.66 (dd, *J* = 17.5, 10.7 Hz, 1H), 6.59 (d, *J* = 2.2 Hz, 2H), 6.41 (t, *J* = 2.2 Hz, 1H), 5.75 (dd, *J* = 17.6, 1.0 Hz, 1H), 5.26 (dd, *J* = 10.9, 1.0 Hz, 1H), 3.80 (s, 6H, 2 × OCH_3_); ^13^C NMR (CDCl_3_, 100 MHz): 160.8, 139.5, 136.8, 114.2, 104.2, 99.9, 55.1 (2 × OCH_3_).

1-methoxy-2-vinylbenzene, **3g**. ^1^H NMR (CDCl_3_, 400 MHz) δ: 7.46 (dd, *J* = 7.8, 1.8 Hz, 1H), 7.23 (t, *J* = 7.8 Hz, 1H), 7.04 (dd, *J* = 17.8, 11.4 Hz, 1H), 6.93 (t, *J* = 7.3 Hz, 1H), 6.87 (d, *J* = 8.2 Hz, 1H), 5.73 (dd, *J* = 17.8, 1.4 Hz, 1H), 5.25 (dd, *J* = 9.1, 1.4 Hz, 1H), 3.84 (s, 3H, OCH_3_); ^13^C NMR (CDCl_3_, 100 MHz): 156.9, 131.9, 129.0, 126.9, 126.7, 120.8, 114.6, 111.0, 55.6.

1-methoxy-3-vinylbenzene, **3h**. ^1^H NMR (CDCl_3_, 400 MHz) δ: 7.26 (t, *J* = 8.0 Hz, 1H), 7.03 (t, *J* = 7.8 Hz, 1H), 6.98 (s, 1H), 6.83 (dd, *J* = 8.2 Hz, 2.8Hz, 1H), 6.71 (dd, *J* = 17.8 Hz, 11.0 Hz, 1H), 5.77 (d, *J* = 17.8 Hz, 1H), 5.27 (d, *J* = 11.0 Hz, 1H), 3.83 (s, OCH_3_, 3H); ^13^C NMR (CDCl_3_, 100 MHz): 159.9, 139.1, 136.9, 129.6, 119.1, 114.2, 113.5, 111.6, 55.3 (OCH_3_).

4-vinyl-1,1′-biphenyl, **3k**. ^1^H NMR (CDCl_3_, 400 MHz) δ: 7.60 (d, *J* = 6.8 Hz, 2H), 7.57 (d, *J* = 8.2 Hz, 2H), 7.49 (d, *J* = 8.7 Hz, 2H), 7.44 (t, *J* = 7.5 Hz, 2H), 7.34 (t, *J* = 7.3 Hz, 1H), 6.76 (dd, *J* = 17.8, 11.0 Hz, 1H), 5.80 (d, *J* = 17.8 Hz, 1H), 5.28 (d, *J* = 11.0 Hz, 1H); ^13^C NMR (CDCl_3_, 100 MHz): 140.9, 140.7, 136.7, 136.5, 128.9, 127.5, 127.4, 127.1, 126.8, 114.0.

#### 3.4.2. General Procedure for the Preparation of *N*-(2-iodophenyl)acylamide

As described in [18] a solution of an appropriate acyl chloride (1 equiv) in 5 mL of dry THF was added dropwise to a stirred, cooled (0–5 °C) solution of 2-iodoaniline (1 equiv) and Et_3_N (1 equiv) in 20 mL of dry THF. The ice bath was then removed and the mixture stirred vigorously overnight at room temperature. Solid Et_3_N·HCl was then filtered off and washed with THF (3 × 5 mL). The resulting organic fractions were combined and THF was removed under reduced pressure to yield crude amides. Recrystallization from hexanes/chloroform and drying in vacuum produced the desired product. The procedure for compounds **5a**–**5f** is attached in Appendix.

*N*-(2-iodophenyl)acetamide, **5a**. *Mp* 103–105 °C. ^1^H NMR (CDCl_3_, 400 MHz) δ: 8.17 (d, *J* = 7.6 Hz, 1H), 7.75 (d, *J* = 7.8 Hz, 1H), 7.40 (br s, 1H, NH), 7.32 (t, *J* = 7.3 Hz, 1H), 6.82 (t, *J* = 7.4 Hz, 1H), 2.22 (s, 3H, CH_3_); ^13^C NMR (CDCl_3_, 100 MHz): 168.1, 138.8, 138.2, 129.2, 126.0, 122.1, 90.0, 24.8 (CH_3_).

*N*-(2-iodophenyl)furan-2-carboxamide, **5b**. *Mp* 80–81 °C; IR (NaCl): 3364, 1683, 1582, 1526, 1430, 1304, 1162, 1010, 750 cm^−1^; UV (MeOH)_max_ nm: 257, 231; ^1^H NMR (CDCl_3_, 400 MHz) δ: 8.52 (br s, 1H, NH,), 8.39 (dd, *J* = 8.7, 1.4 Hz, 1H), 7.80 (dd, *J* = 7.8, 1.4 Hz, 1H), 7.56 (d, *J* = 1.4 Hz, 1H), 7.36 (td, *J* = 8.7, 1.4 Hz, 1H), 7.26 (d, *J* = 3.7 Hz, 1H), 6.85 (td, *J* = 7.8, 1.4 Hz, 1H), 6.57 (dd, *J* = 3.6, 1.8 Hz, 1H); ^13^C NMR (CDCl_3_, 100 MHz): 156.1, 147.7, 144.8, 139.0, 138.0, 129.4, 126.1, 121.7, 115.8, 112.8, 89.9; HRMS (+ESI) [M + H]^+^: 313.9671, C_11_H_9_INO_2_ requires 313.9672.

*N*-(2-iodophenyl)benzamide, **5c**. *Mp* 135–137 °C; ^1^H NMR (CDCl_3_, 400 MHz) δ: 8.46 (d, *J* = 8.3 Hz, 1H), 8.30 (br s, 1H, NH), 7.96–7.98 (m, 2H), 7.82 (dd, *J* = 7.9, 1.5 Hz, 1H), 7.51–7.61 (m, 3H), 7.41 (td, *J* = 7.8, 1.5 Hz, 1H), 6.89 (td, *J* = 7.7, 1.5 Hz, 1H); ^13^C NMR (CDCl_3_, 100 MHz): 165.4, 138.9, 138.4, 134.6, 132.3, 129.5, 129.0, 127.3, 126.2, 121.9, 90.3.

*N*-(2-iodophenyl)cyclohexanecarboxamide, **5d**. *Mp* 134–146 °C; ^1^H NMR (CDCl_3_, 400 MHz) δ: 8.24 (d, *J* = 8.2 Hz, 1H), 7.75 (dd, *J* = 8.2, 1.4 Hz, 1H), 7.51 (br s, 1H, NH), 7.32 (td, *J* = 7.4, 1.4 Hz, 1H), 6.82 (td, *J* = 7.8, 1.8 Hz, 1H), 2.30 (tt, *J* = 11.7, 3.4 Hz, 1H), 1.23–2.06 (m, 10H); ^13^C NMR (CDCl_3_, 100 MHz): 174.4, 138.8, 138.3, 129.3, 125.9, 122.1, 90.2, 46.7, 29.8 (CH_2_), 25.8 (CH_2_).

*N*-(2-iodophenyl)isobutyramide, **5e**. *Mp* 110–111 °C; ^1^H NMR (CDCl_3_, 400 MHz) δ: 8.23 (d, *J* = 8.0 Hz, 1H,), 7.75 (d, *J* = 8.1 Hz, 1H), 7.51 (br s, 1H, NH), 7.32 (t, *J* = 7.6 Hz, 1H), 6.82 (t, *J* = 7.3 Hz, 1H), 2.59 (septet, *J* = 7.0 Hz, 1H), 1.30 (d, *J* = 6.8 Hz, 6H, 2 × CH_3_); ^13^C NMR (CDCl_3_, 100 MHz): 175.3, 138.8, 138.2, 129.4, 125.9, 122.0, 90.2, 37.1, 19.7 (CH_3_).

*N*-(2-iodophenyl)butyramide, **5f**. *Mp* 81–83 °C; ^1^H NMR (CDCl_3_, 400 MHz) δ: 8.22 (d, *J* = 7.8 Hz, 1H), 7.76 (dd, *J* = 8.0, 1.4 Hz, 1H), 7.43 (br s, 1H, NH,), 7.33 (td, *J* = 8.0, 1.4 Hz, 1H), 6.82 (t, *J* = 7.8 Hz, 1H), 2.40 (d, *J* = 7.6 Hz, 2H), 1.79 (sextet, *J* = 7.8 Hz, 2H), 1.03 (t, *J* = 7.3 Hz, 3H, CH_3_); ^13^C NMR (CDCl_3_, 100 MHz): 171.3, 138.8, 138.3, 129.4, 126.0, 122.1, 90.0, 40.0, 19.2, 13.7 (CH_3_).

#### 3.4.3. General Procedure for Stilbene

In a dry two neck flask, the appropriate *N*-(2-iodophenyl)acylamide (1 equiv) was dissolved in dry DMF and stirred under nitrogen. The solution was heated up to 120 °C and refluxed for a few minutes. Palladium (II) acetate (0.01 equiv) was added, followed by triethylamine (3.5 equiv) into the mixture. Lastly, the appropriate styrene (1.2 equiv) was added into the reaction flask. The mixture was heated at 120 °C under nitrogen until consumption of *N*-(2-iodophenyl)acylamide (check via TLC). The reaction mixture was quenched with saturated ammonium chloride aqueous solution. It was then extracted with ethyl acetate and washed with plenty of distilled water. The resulting organic fractions were combined, dried over anhydrous sodium sulphate and solvent was removed under reduced pressure to yield crude product. Purification by column chromatography afforded the desired products. The procedure for compounds **6a**–**6s** is attached in Appendix.

(*E*)*-N*-(2-(4-chlorostyryl)phenyl)acetamide, **6a**[22]. *Mp* 201–202 °C; IR (neat): 3281, 1642, 1531, 1299, 951, 803, 748 cm^−1; 1^H NMR (CDCl_3_, 400 MHz) δ: 7.42 (d, *J* = 8.3 Hz, 2H), 7.33 (d, *J* = 8.5 Hz, 2H), 6.95 (d, *J* = 16.0 Hz, 1H), 7.11 (d, *J* = 16.0 Hz, 1H), 7.75 (d, *J* = 8.2 Hz, 1H), 7.18 (t, *J* = 7.3 Hz, 1H), 7.52 (d, *J* = 7.3 Hz, 1H), 2.21 (s, 3H, CH_3_); ^13^C NMR (CDCl_3_, 100 MHz) 135.5, 127.8, 128.9, 133.7, 128.9, 127.8, 130.9, 124.1, 130.4, 134.6, 124.7, 128.5, 125.8, 126.7, 24.2, 168.6 (C=O); HRMS (+ESI) [M + H]^+^: 272.0873, C_16_H_15_ClNO requires 272.0842.

(*E*)*-N*-(2-(3-chlorostyryl)phenyl)acetamide, **6b**. IR (neat): 3281, 1655, 1530, 1298, 951, 773, 749 cm^−1; 1^H NMR (CDCl_3_, 400 MHz) δ: 7.47 (br s, 1H), 7.35–7.24 (m, 4H), 6.92 (d, *J* = 16.0 Hz, 1H), 7.10 (d, *J* = 16.0 Hz, 1H), 7.73 (d, *J* = 8.2 Hz, 1H), 7.18 (d, *J* = 7.8 Hz, 1H), 7.52 (d, *J* = 7.8 Hz, 1H), 2.21 (s, 3H, CH_3_); ^13^C NMR (CDCl_3_, 100 MHz) 139.0, 126.9, 134.8, 125.0, 128.8, 130.1, 130.8, 125.1, 130.3, 134.8, 124.8, 128.1, 125.9, 126.5, 24.4 (CH_3_), 168.8 (C=O); HRMS (+ESI) [M + H]^+^: 272.0800, C_16_H_15_ClNO requires 272.0842.

(*E*)*-N*-(2-(4-fluorostyryl)phenyl)acetamide, **6c**. IR (neat): 3259, 1659, 1527, 1297, 963, 818 cm^−1; 1^H NMR (CDCl_3_, 400 MHz) δ: 7.46 (d, *J* = 6.0 Hz, 2H), 7.01 (d, *J* = 8.2 Hz, 2H), 6.95 (d, *J* = 16.5 Hz, 1H), 7.01–7.05 (m, 1H), 7.73 (d, *J* = 8.2 Hz, 1H), 7.26 (t, *J* = 6.8 Hz, 1H), 7.19 (t, *J* = 7.8 Hz, 1H), 7.51 (d, *J* = 7.8 Hz, 1H), 2.21 (s, 3H, CH_3_); ^13^C NMR (CDCl_3_, 100 MHz) 163.7, 128.1, 115.7, 161.2, 115.5, 128.1, 131.0, 123.2, 130.4, 134.4, 124.5, 128.2, 125.6, 126.6, 24.1 (CH_3_), 168.6 (C=O); HRMS (+ESI) [M + H]^+^: 256.1139, C_16_H_15_FNO requires 256.1138.

(*E*)*-N*-(2-(3-fluorostyryl)phenyl)acetamide, **6d**. IR (neat): 3281, 1642, 1531, 1299, 951, 803, 748 cm^−1; 1^H NMR (CDCl_3_, 400 MHz) δ: 7.14 (br s, 1H), 6.94 (td, *J* = 1.4, 8.2 Hz, 1H), 7.29 (t, *J* = 7.4 Hz, 1H), 7.16–7.05 (m, 3H), 6.81 (d, *J* = 16.5 Hz, 1H), 7.04–7.10 (m, 1H), 7.48 (d, *J* = 7.8 Hz, 1H), 7.43 (d, *J* = 7.8 Hz, 1H), 2.10 (s, 3H, CH_3_); ^13^C NMR (CDCl_3_, 100 MHz) 139.6, 112.9, 162.0, 113.1, 130.2, 122.8, 130.3, 125.3, 130.4, 134.6, 124.7, 128.5, 125.8, 126.7, 24.2 (CH_3_), 168.6; HRMS (+ESI) [M + H]^+^: 256.1810, C_16_H_15_FNO requires 256.1138.

(*E*)*-N*-(2-(4-bromostyryl)phenyl)acetamide, **6e**. IR (neat): 3315, 1664, 1557, 1259, 1029, 798, 772 cm^−1; 1^H NMR (CDCl_3_, 400 MHz) δ: 7.48 (d, *J* = 8.2 Hz, 2H), 7.34 (d, *J* = 8.2 Hz, 2H), 6.91 (d, *J* = 16.5 Hz, 1H), 7.12 (d, *J* = 16.5 Hz, 1H), 7.70 (d, *J* = 7.8 Hz, 1H), 7.27 (d, *J* = 7.8 Hz, 1H), 7.17 (t, *J* = 7.8 Hz, 1H), 7.52 (d, *J* = 7.8 Hz, 1H), 2.19 (s, 3H, CH_3_); ^13^C NMR (CDCl_3_, 100 MHz) 136.1, 132.0, 128.2, 121.9, 128.2, 132.0, 130.9, 124.3, 130.5, 134.7, 124.9, 128.6, 126.0, 126.8, 24.3 (CH_3_), 168.9; HRMS (+ESI) [M + H]^+^: 316.0334, C_16_H_15_BrNO requires 316.0377.

(*E*)*-N*-(2-(3,5-dimethoxystyryl)phenyl)acetamide, **6f**. IR (NaCl): 3262, 1654, 1153, 773 cm^−1^. UV (MeOH)_max_ nm: 305, 215; ^1^H NMR (CDCl_3_, 400 MHz) δ: 7.77 (d, *J* = 8.0 Hz, 1H), 7.49 (d *J* = 8.0 Hz, 1H), 7.27 (t, *J* = 8.0 Hz, 1H), 7.20 (br s, 1H, NH), 7.15 (t, *J* = 8.0 Hz, 1H), 7.08 (d, *J* = 16.1 Hz, 1H), 6.88 (d, *J* = 16.1 Hz, 1H), 6.63 (d, *J* = 2.2 Hz, 2H), 6.41 (t, *J* = 2.2 Hz, 1H), 3.82 (s, 6H, OCH_3_), 2.19 (s, 3H, CH_3_). ^13^C NMR (CDCl_3_, 100 MHz): 168.8, 161.1, 139.1, 134.7, 132.4, 130.3, 128.4, 126.9, 125.7, 124.4, 124.2, 105.0, 100.1, 55.4, 24.3; HRMS (+ESI) [M + H]^+^: 298.1430, C_18_H_20_NO_3_ requires 298.1443.

(*E*)-*N*-(2-(2-methoxystyryl)phenyl)furan-2-carboxamide, **6g**[15]. *Mp* 128–129 °C; IR (NaCl): 3285, 1671, 1585, 1304, 1247, 751 cm^−1^; UV (MeOH)_max_ nm: 262, 321; ^1^H NMR (CDCl_3_, 400 MHz) δ: 8.25 (br s, 1H, NH), 8.04 (d, *J* = 8.2 Hz, 1H), 7.60 (dd, *J* = 7.8, 1.0 Hz, 1H), 7.56 (dd, *J* = 7.6, 1.4 Hz, 1H), 7.49 (d, *J* = 1.0 Hz, 1H), 7.40 (d, *J* = 16.5 Hz, 1H), 7.29 (d, *J* = 16.8 Hz, 1H), 7.28–7.33 (m, 3H), 7.20 (t, *J* = 7.1 Hz, 1H), 6.98 (t, *J* = 7.6 Hz, 1H), 6.91 (d, *J* = 8.2 Hz, 1H), 6.55 (dd, *J* = 3.7, 1.8 Hz, 1H), 3.85 (s, 3H OCH_3_); ^13^C NMR (CDCl_3_, 100 MHz): 157.3, 156.4, 148.0, 144.5, 134.1, 130.7, 129.3, 128.3, 128.2, 127.3, 127.2, 126.2, 125.6, 123.7, 123.5, 120.9, 115.4, 112.7, 111.1, 55.5 (OCH_3_); HRMS (+ESI) [M + H]^+^: 320.1285, C_20_H_18_NO_3_ requires 320.1287.

(*E*)-*N*-(2-(3-methoxystyryl)phenyl)furan-2-carboxamide, **6h**[15]. *Mp* 96–97 °C; IR (NaCl): 3285, 1669, 1585, 1521, 1304, 1163, 755 cm^−1^; UV (MeOH)_max_ nm: 210, 264, 300; ^1^H NMR (CDCl_3_, 400 MHz) δ: 8.17 (br s, 1H, NH), 8.02 (d, *J* = 8.2 Hz, 1H), 7.55 (d, *J* = 7.8 Hz, 1H), 7.50 (d, *J* = 1.0 Hz, 1H), 7.33 (t, *J* = 7.2 Hz, 1H), 7.29 (t, *J* = 8.0 Hz, 1H), 7.26 (d, *J* = 3.2 Hz, 1H), 7.22 (d, *J* = 16.0 Hz, 1H), 7.21 (t, *J* = 7.6 Hz, 1H), 7.12 (d, *J* = 7.8 Hz, 1H), 7.04 (s, 1H), 7.02 (d, *J* = 16.5 Hz, 1H), 6.85 (dd, *J* = 8.5, 2.3 Hz, 1H), 6.56 (dd, *J* = 3.7, 1.8 Hz, 1H), 3.83 (s, 3H, OCH_3_); ^13^C NMR (CDCl_3_, 100 MHz): 160.0, 156.4, 147.9, 144.5, 138.5, 134.2, 132.9, 130.1, 129.9, 128.6, 127.3, 125.6, 123.6, 119.4, 115.6, 113.7, 112.7, 112.3, 55.4 (OCH_3_); HRMS (+ESI) [M + H]^+^: 320.1282, C_20_H_18_NO_3_ requires 320.1287.

(*E*)-*N*-(2-(4-methoxystyryl)phenyl)furan-2-carboxamide, **6i**[15]. *Mp* 120–121 °C; IR (NaCl): 3276, 1669, 1584, 1509, 1251, 755 cm^−1^; UV (MeOH)_max_ nm: 271, 323; ^1^H NMR (CDCl_3_, 400 MHz) δ: 8.17 (br s, 1H, NH), 8.04 (d, *J* = 8.2 Hz, 1H), 7.54 (d, *J* = 7.8 Hz, 1H), 7.50 (d, *J* = 1.0 Hz, 1H), 7.47 (t, *J* = 7.6 Hz, 2H), 7.31 (td, *J* = 7.3, 1.3 Hz, 1H), 7.26 (d, *J* = 3.7 Hz, 1H), 7.19 (t, *J* = 7.6 Hz, 1H), 7.09 (d, *J* = 16.5 Hz, 1H), 7.00 (d, *J* = 16.5 Hz, 1H), 6.92 (t, *J* = 7.6 Hz, 2H), 6.56 (dd, *J* = 3.2, 1.8 Hz, 1H), 3.83 (s, 3H, OCH_3_); ^13^C NMR (CDCl_3_, 100 MHz): 159.8, 156.3, 148.0, 144.5, 134.0, 132.7, 130.4, 129.9, 128.2, 128.1, 127.1, 125.6, 123.5, 121.0, 115.5, 114.3, 112.7, 55.5 (OCH_3_); HRMS (+ESI) [M + H]^+^: 320.1302, C_20_H_18_NO_3_ requires 320.1287.

(*E*)-*N*-(2-(3,4-dimethoxystyryl)phenyl)furan-2-carboxamide, **6j**[15]. *Mp* 142–144 °C; IR (NaCl): 3239, 1651, 1575, 1514, 1456, 1265, 1157, 1137, 1027, 751 cm^−1^; UV (MeOH)_max_ nm: 210, 252, 326; ^1^H NMR (CDCl_3_, 400 MHz) δ: 8.19 (br s, 1H, NH), 8.00 (d, *J* = 8.2 Hz, 1H), 7.52 (d, *J* = 7.8 Hz, 1H), 7.47 (dd, *J* = 1.8, 1.0 Hz, 1H), 7.29 (t, *J* = 7.8 Hz, 1H), 7.23 (d, *J* = 2.8 Hz, 1H), 7.18 (t, *J* = 7.8 Hz, 1H), 7.07 (d, *J* = 16.5 Hz, 1H), 7.05 (d, *J* = 8.9 Hz, 1H), 7.03 (s, 1H), 6.96 (d, *J* = 16.5 Hz, 1H), 6.85 (d, *J* = 8.2, 2.3 Hz, 1H), 6.54 (dd, *J* = 3.4, 1.8 Hz, 1H), 3.89 (s, 3H, OCH_3_), 3.88 (s, 3H, OCH_3_); ^13^C NMR (CDCl_3_, 100 MHz): 156.4, 149.4, 149.2, 148.0, 144.5, 134.0, 132.7, 130.4, 130.2, 128.2, 127.1, 125.6, 123.7, 121.4, 120.1, 115.4, 112.7, 111.4, 109.3, 56.1 (OCH_3_), 56.0 (OCH_3_); HRMS (+ESI) [M + H]^+^: 320.1411, C_21_H_20_NO_4_ requires 320.1392.

(*E*)*-N*-(2-(3,5-dimethoxystyryl)phenyl)furan-2-carboxamide, **6k**[15]. *Mp* 109–110 °C; IR (NaCl): 3282, 1670, 1590, 1520, 1455, 1303, 1204, 1152, 1065, 755 cm^−1^; UV (MeOH)_max_ nm: 239, 269, 308; ^1^H NMR (CDCl_3_, 400 MHz) δ: 8.16 (br s, 1H, NH), 8.04 (d, *J* = 7.8 Hz, 1H), 7.54 (dd, *J* = 7.8, 1.4 Hz, 1H), 7.50 (d, *J* = 1.0 Hz, 1H), 7.33 (td, *J* = 7.8, 1.4 Hz, 1H), 7.26 (d, *J* = 3.2 Hz, 1H), 7.21 (d, *J* = 16.0 Hz, 1H), 7.20 (t, *J* = 7.5 Hz, 1H), 6.99 (d, *J* = 16.0 Hz, 1H), 6.66 (s, 2H, H-2), 6.56 (dd, *J* = 3.6, 1.8 Hz, 1H), 6.43 (t, *J* = 1.8 Hz, 1H), 3.81 (s, 6H, 2xOCH_3_); ^13^C NMR (CDCl_3_, 100 MHz): 161.1, 156.3, 147.9, 144.5, 139.1, 134.2, 133.0, 129.9, 128.6, 127.3, 125.6, 123.9, 123.6, 115.6, 112.7, 105.0, 100.3, 55.9 (2 × OCH_3_); HRMS (+ESI) [M + H]^+^: 350.1412, C_21_H_20_NO_4_ requires 350.1392.

(*E*)-*N*-(2-(2-(biphenyl-4-yl)vinyl)phenyl)furan-2-carboxamide, **6l**[15]. *Mp* 142–143 °C; IR (NaCl): 3283, 1671, 1585, 1521, 1487, 1452, 1304, 762 cm^−1^; UV (MeOH)_max_ nm: 204, 267, 323; ^1^H NMR (CDCl_3_, 400 MHz) δ: 8.19 (br s, 1H, NH), 8.04 (d, *J* = 7.8 Hz, 1H), 7.58–7.64 (m, 7H), 7.52 (t, *J* = 1.0 Hz, 1H), 7.44–7.48 (m, 2H), 7.32–7.38 (m, 2H), 7.21–7.30 (m, 3H), 7.10 (d, *J* = 16.5 Hz, 1H); ^13^C NMR (CDCl_3_, 100 MHz): 156.4, 148.0, 144.5, 141.0, 140.6, 136.1, 134.2, 132.5, 130.2, 129.0, 128.6, 127.6, 127.5, 127.3, 127.0, 125.7, 123.7, 123.3, 115.6, 112.7; HRMS (+ESI) [M + Na]^+^: 388.1324, C_27_H_22_NO requires 388.1308. An X-ray of this compound was published in 2008 [23].

(*E*)*-N-*(2-(2-([1,1′-biphenyl]-4-yl)vinyl)phenyl)benzamide, **6m**[15]. *Mp* 167–169 °C; IR (NaCl): 3271, 1648, 1517, 1487, 1302, 764 cm^−1^; UV (MeOH)_max_ nm: 319; ^1^H NMR (CDCl_3_, 400 MHz) δ: 8.05 (br s, 1H, NH), 7.91 (d, *J* = 7.3 Hz, 3H), 7.43–7.62 (m, 12H), 7.31–7.36 (m, 2H), 7.22–7.26 (m, 2H), 7.06 (d, *J* = 16.5 Hz, 1H); ^13^C NMR (CDCl_3_, 100 MHz): 166.0, 140.9, 140.6, 136.1, 134.8, 134.7, 132.2, 132.1, 131.0, 129.0, 128.5, 127.5, 127.3, 127.2, 127.1, 127.0, 126.0, 124.7, 123.5; HRMS (+ESI) [M + H]^+^: 376.1691, C_27_H_22_NO requires 376.1701.

(*E*)-*N*-(2-(3,4-dimethoxystyryl)phenyl)benzamide, **6n**[15]. *Mp* 169–171 °C; IR (NaCl): 3273, 3012, 1646, 1572, 1509, 1265, 749 cm^−1^; UV (MeOH)_max_ nm: 231, 324; ^1^H NMR (CDCl_3_, 400 MHz) δ: 8.03 (br s, 1H, NH), 7.87 (d, *J* = 7.8 Hz, 3H), 7.51–7.55 (m, 2H), 7.44 (t, *J* = 7.3 Hz, 2H), 7.28 (t, *J* = 7.8 Hz, 2H), 7.20 (t, *J* = 7.5 Hz, 1H), 7.04 (d, *J* = 16.0 Hz, 1H), 6.98 (d, *J* = 9.2 Hz, 1H), 6.97 (s, 1H), 6.93 (d, *J* = 16.0 Hz, 1H), 6.82 (d, *J* = 8.2 Hz, 1H), 3.86 (s, 3H, OCH_3_), 3.83 (s, 3H, OCH_3_); ^13^C NMR (CDCl_3_, 100 MHz): 166.0, 149.4, 149.2, 134.8, 134.7, 132.5, 132.0, 131.1, 130.2, 128.9, 128.2, 127.3, 127.0, 125.9, 124.5, 121.7, 120.1, 111.3, 109.1, 56.0 (OCH_3_), 55.9 (OCH_3_); HRMS (+ESI) [M + H]^+^: 360.1594, C_23_H_22_NO_3_ requires 360.1600.

(*E*)*-N*-(2-(4-methoxystyryl)phenyl)benzamide, **6o**. IR (neat): 3283, 1644, 1512, 1482, 1248, 1178, 1027, 749 cm^−1; 1^H NMR (400MHz, CDCl_3_) δ: 3.82 (s, 3H. OCH_3_), 6.87 (dt, *J* = 3.2, 8.7 Hz, 2H), 6.98 (d, *J* = 16.0 Hz, 1H), 7.06 (d, *J* = 16.5 Hz, 1H), 7.21 (t, *J* = 7.6 Hz, 1H), 7.32 (t, *J* = 7.8 Hz, 1H), 7.41 (t, *J* = 4.3 Hz, 2H), 7.48 (t, *J* = 7.6 Hz, 2H), 7.54 (t, *J* = 7.3 Hz 2H), 7.89 (d, *J* = 7.3 Hz, 2H), 7.97 (d, *J* = 7.8 Hz, 1H); ^13^C NMR (CDCl_3_, 100 MHz): 132.0, 129.0, 127.2, 134.8, 134.6, 124.1, 128.2, 125.8, 127.1, 130.9, 121.1, 132.9, 129.8, 128.1, 114.3, 159.8, 165.5; HRMS (+ESI) [M + H]^+^: 330.1493, C_22_H_20_NO_2_ requires 330.1494.

(*E*)-*N*-(2-(3,4-dimethoxystyryl)phenyl)cyclohexanecarboxamide, **6p**[15]. *Mp* 204–206 °C; IR (NaCl): 2930, 2853, 1682, 1515, 1449, 1269, 1137, 1026, 757 cm^−1^; UV (MeOH)_max_ nm: 217, 300; ^1^H NMR (CDCl_3_, 400 MHz) δ: 7.77 (d, *J* = 8.2 Hz, 1H), 7.50 (d, *J* = 7.3 Hz, 1H), 7.25 (td, *J* = 8.0, 1.4 Hz, 1H), 7.21 (br s, 1H, NH), 7.17 (d, *J* = 7.8 Hz, 1H), 7.16 (d, *J* = 7.6 Hz, 1H), 7.03 (d, *J* = 7.3 Hz, 1H), 7.02 (s, 1H), 6.97 (d, *J* = 16.5 Hz, 1H), 6.91 (d, *J* = 16.5 Hz, 1H), 3.91 (s, 3H, OCH_3_), 3.90 (s, 3H, OCH_3_), 2.31 (tt, *J* = 11.7, 3.4 Hz, 1H), 1.22–2.01 (m, 10H);^13^C NMR (CDCl_3_, 100 MHz): 174.5, 149.3, 149.2, 134.6, 132.2, 130.8, 130.2, 128.1, 126.8, 125.6, 124.4, 121.7, 120.1, 111.3, 108.9, 56.1 (OCH_3_), 55.9 (OCH_3_), 46.3, 29.9, 25.8; HRMS (+ESI) [M + H]^+^: 366.2069, C_23_H_28_NO_3_ requires 366.2069.

(*E*)-*N*-(2-(3,4-dimethoxystyryl)phenyl)isobutyramide, **6q**[15]. *Mp* 166–168 °C; IR (NaCl): 3274, 1652, 1516, 1269, 1141, 1025, 961, 801, 752 cm^−1^; UV (MeOH)_max_ nm: 210, 236, 325; ^1^H NMR (CDCl_3_, 400 MHz) δ: 7.74 (d, *J* = 7.8 Hz, 1H), 7.49 (d, *J* = 7.3 Hz, 1H), 7.31 (br s, 1H, NH), 7.24 (t, *J* = 7.8 Hz, 1H), 7.15 (t, *J* = 7.6 Hz, 1H), 7.01 (d, *J* = 7.8 Hz, 1H), 7.00 (s, 1H), 6.97 (d, *J* = 16.4 Hz, 1H), 6.90 (d, *J* = 16.5 Hz, 1H), 6.85 (d, *J* = 8.7 Hz, 1H), 3.89 (s, 6H, 2xOCH_3_), 2.54–2.61 (m, 1H), 1.27 (d, *J* = 6.9 Hz, 6H); ^13^C NMR (CDCl_3_, 100 MHz): 175.6, 149.3, 149.2, 134.6, 132.1, 130.9, 130.2, 128.1, 126.8, 125.7, 124.5, 121.7, 120.1, 111.3, 108.9, 56.1 (OCH_3_), 55.9 (OCH_3_), 36.5, 19.9 (2 × CH_3_); HRMS (+ESI) [M + H]^+^: 326.1759, C_20_H_24_NO_3_ requires 326.1756.

(*E*)*-N*-(2-(4-methoxystyryl)phenyl)isobutyramide, **6r**. IR (NaCl): 3267, 2966, 1510, 1267, 1033, 750 cm^−1; 1^H NMR (400MHz, CDCl_3_) δ: 1.27 (d, *J* = 6.9 Hz, 6H), 2.53–2.59 (m, 1H), 3.82 (s, 3H, OCH_3_), 7.79 (d, *J* = 7.8 Hz, 1H), 7.23 (td, *J* = 1.0, 8.0 Hz, 1H), 7.14 (d, *J* = 7.5 Hz, 1H), 7.48 (d, *J* = 7.8 Hz, 1H), 6.97 (d, *J* = 16.5 Hz, 1H), 6.92 (m, 1H), 7.41 (d, *J* = 9.2 Hz, 2H), 6.89 (m, 2H), 7.33 (br s, 1H, NH); ^13^C NMR (CDCl_3_, 100 MHz): 19.8 (CH_3_), 36.5, 55.4 (OCH_3_), 134.6, 124.2, 128.0, 125.5, 126.9, 130.7, 121.3, 132.0, 129.9, 128.0, 114.3, 175.5; HRMS (+ESI) [M + H]^+^: 296.1649, C_19_H_22_NO_2_ requires 296.1651.

(*E*)-*N*-(2-(3,4-dimethoxystyryl)phenyl)butyramide, **6s**[15]. *Mp* 139–140 °C; IR (NaCl): 3280, 2963, 1510, 1267, 1025, 759 cm^−1^; UV (MeOH)_max_ nm: 206, 323; ^1^H NMR (CDCl_3_, 400 MHz) δ: 7.63 (d, *J* = 7.8 Hz, 1H), 7.56 (br s, 1H, NH), 7.46 (d, *J* = 7.8 Hz, 1H), 7.18 (t, *J* = 7.0 Hz, 1H), 7.11 (t, *J* = 7.3 Hz, 1H), 6.98 (d, *J* = 7.8 Hz, 1H), 6.97 (s, 1H), 6.95 (d, *J* = 15.6 Hz, 1H), 6.85 (d, *J* = 16.2 Hz, 1H), 6.81 (d, *J* = 8.2 Hz, 1H), 3.86 (s, 3H, OCH_3_), 3.85 (s, 3H, OCH_3_), 2.31 (t, *J* = 7.3 Hz, 2H), 1.69–1.75 (m, 2H, CH_2_), 0.97 (t, *J* = 7.3 Hz, 3H); ^13^C NMR (CDCl_3_, 100 MHz): 172.0, 149.2, 149.1, 134.6, 131.6, 131.1, 130.3, 127.9, 126.5, 125.7, 124.9, 121.8, 120.1, 111.3, 109.1, 56.0 (OCH_3_), 55.9 (OCH_3_), 39.3, 19.4, 13.9 (CH_3_); HRMS (+ESI) [M + H]^+^: 326.1755, C_20_H_24_NO_3_ requires 326.1756.

### 3.5. Cytotoxic Assays

Cytotoxicity of the compounds were evaluated against a panel of 5 cancer cell lines; prostate (DU-145), pancreatic (BxPC-3), colon (HT-29), breast (MCF-7) and (MDA-MB-231); and one normal cell line, pancreatic (hTERT-HPNE). The cancer cell lines were chosen from the National Cancer Institute (NCI) list of 60 cancer cell lines for drug screening and drug treatment conditions adhered to NCI recommendations [24].

Cell lines were cultured in DMEM media supplemented with 2 mM l-glutamine, 10% foetal bovine serum, 50 μg/mL gentamicin and 2.5 μg/mL amphotericin B, maintained in a 37 °C humid atmosphere of 5% CO_2_ cell incubator. hTERT-HPNE cell line was cultured in the same DMEM with 10 ng/mL human recombinant epithelial growth factor as recommended by ATCC. Samples and drug standards (cisplatin, resveratrol and vinblastine) were dissolved in DMSO and immediately diluted with DMEM media to yield a final DMSO concentration of less than 0.5% *v*/*v*.

Cells were plated into 96-well microplates at 5000–10,000 cells per well and maintained in the cell incubator for 24 h. Then, 100 μL of samples were introduced in triplicates to a final concentration of 0.1–200 μM. Drug standards were also introduced to a final concentration of 0.03–2000 μM (cisplatin) 0.78–400 μM (resveratrol) and 0.002–200 μM (vinblastine). Cells were further incubated for 48 h and then, cell viability was determined according to the manufacturer protocol of a commercial MTS assay kit (CellTiter 96^®^ AQ_ueous_ One Solution, Promega). Culture media were carefully refreshed with 100 μL of DMEM media, followed by 20 μL per well of MTS reagent. Microplates were returned to the incubator for 1–2 h and absorbance of the formazan product was read on a microplate reader at 490 nm with 690 nm as the background wavelength (Infinite 200, Tecan, Mannedorf, Swizerland). IC_50_ of samples and drug standards were determined using dose-response curves, and statistical analysis using student’s *T*-test (*p* < 0.05) was performed in Prism 5.02 software (GraphPad Software Inc., La Jolla, CA, USA).

## 4. Conclusions

A total of 19 analogues of *o*-carboxamido stilbenes were synthesized using the Heck method. These analogues possessing the amide moiety with different substituent groups displayed varying cytotoxic activity toward various human cancer cell lines with the most potent compounds; compound **6d** (IC_50_ = 16.68 μM, prostate cancer DU-145), compound **6i** (IC_50_ = 7.51 μM, colon cancer HT-29), compound **6n** (IC_50_ = 21.24 μM, estrogen-sensitive breast cancer MCF-7) and compound **6s** (IC_50_ = 66.30 μM, pancreatic cancer BxPC-3). Interestingly, all these potent compounds did not show any cytotoxicity toward the normal pancreatic cell line (hTERT-HPNE) compared to vinblastine, cisplastin and resveratrol which exhibited cytotoxicity toward the normal pancreatic cell line (hTERT-HPNE). These results provide progress in our knowledge to support future designs of stilbene-based anticancer drugs.

## Figures and Tables

**Table 1.** Cytotoxic evaluation of *o*-carboxamido stilbenes on human cancer cell lines.

Entry	IC_50_/μM (*n* = 3)			

	HT-29	MCF-7	Bx-PC-3	DU-145
Vinblastin (Positive control)	4.40 ± 2.70	21.00 ± 2.33	33.04 ± 2.14	20.31 ± 7.76
Cisplatin (Positive control)	30.61 ± 2.34	55.04 ± 5.25	71.54 ± 5.36	22.68 ± 1.14
Resveratrol (Control)	72.9 ± 2.4	85.71 ± 1.70	71.85 ± 1.55	107.92 ± 1.57
**6a**	>200	97.00 ± 4.79	>200	>200
**6b**	>200	>200	>200	>200
**6c**	>200	>200	>200	>200
**6d**	11.40 ± 7.35	32.52 ± 5.16	129.78 ± 4.36	16.68 ± 1.86
**6e**	15.92 ± 3.85	>200	>200	>200
**6f**	>200	>200	>200	>200
**6g**	11.06 ± 0.27	>200	>200	>200
**6h**	18.62 ± 0.56	>200	>200	>200
**6i**	7.51 ± 0.53	53.32 ± 5.63	>200	29.19 ± 1.44
**6j**	>200	>200	>200	42.25 ± 2.02
**6k**	15.60 ± 2.10	>200	>200	56.83 ± 2.37
**6l**	>200	115.83 ± 2.90	>200	92.79 ± 4.85
**6m**	>200	>200	>200	>200
**6n**	>200	21.24 ± 1.01	>200	>200
**6o**	>200	>200	>200	>200
**6p**	>200	128.16 ± 7.58	>200	67.89 ± 3.97
**6q**	>200	>200	>200	>200
**6r**	>200	>200	>200	>200
**6s**	29.11 ± 2.73	52.04 ± 7.62	66.30 ± 1.14	>200

## Appendix

### Experimental Data

1,3-methoxy-5-vinylbenzene, **3f**. 3,5-methoxybenzaldehyde **2a** (5.00 g, 30.09 mmol) was treated with methyltriphenylphosphonium iodide (12.37 g, 30.61 mmol) and potassium *tert*-butoxide (3.41 g, 30.35 mmol) according to the general procedure. Purification by column chromatography (hexane/ethyl acetate, 98:2) afforded the product as colourless oil. Yield: 2.68 g (54%).

1-methoxy-2-vinylbenzene, **3g**. 2-methoxybenzaldehyde **2b** (5.04 g, 37.00 mmol) was treated with methyltriphenylphosphonium iodide (14.84 g, 36.72 mmol) and potassium *tert*-butoxide (4.13 g, 36.82 mmol) according to the general procedure. Purification by column chromatography (hexane/ethyl acetate, 98:2) afforded the product as colourless oil. Yield: 2.82 g (57%).

1-methoxy-3-vinylbenzene, **3h**. 3-methoxybenzaldehyde **2c** (5.04 g, 36.76 mmol) was treated with methyltriphenylphosphonium iodide (14.92 g, 36.91 mmol) and potassium *tert*-butoxide (4.24 g, 37.79 mmol) according to the general procedure. Purification by column chromatography (hexane/ethyl acetate, 98:2) afforded the product as colourless oil. Yield: 2.95 g (60 (%).

4-vinyl-1,1′-biphenyl, **3k**. Biphenyl-4-carbaldehyde **2d** (0.99 g, 5.44 mmol) was treated with methyltriphenylphosphonium iodide (2.25 g, 5.56 mmol) and potassium *tert*-butoxide (0.62 g, 5.50 mmol) according to the general procedure. Purification by column chromatography (hexane/ethyl acetate, 98:2) afforded the product as white solid. Yield: 0.54 g (55%).

*N*-(2-iodophenyl)acetamide, **5a**. To a stirred, cooled (0–5 °C) solution of 2-iodoaniline (2.53 g, 11.57 mmol) in 20 mL of dry DMF, sodium hydride (0.92 g, 23.14 mmol) and acetic anhydride (5.40 mL, 61.75 mmol) was added into the reaction flask. Then ice bath was removed and the mixture was allowed to stir overnight at room temperature. Saturated ammonium chloride was added to the reaction mixture followed by extraction with ethyl acetate. The combined ethyl acetate extracts were then washed with distilled water to remove DMF. The solution was then filtered and dried over anhydrous sodium sulphate. The crude product, obtained after evaporation under reduced pressure was purified by column chromatography to give the purified product as yellowish solid. Yield: 1.82 g (60%).

*N*-(2-iodophenyl)furan-2-carboxamide, **5b**. 2-iodoaniline (5.48 g, 25.03 mmol) was treated with furan-2-carbonyl chloride (2.5 mL, 25.40 mmol) according to the general procedure. The crude product was purified by column chromatography (hexane/ethyl acetate, 90:10) to give a yellowish solid. Yield: 5.49 g (69%).

*N*-(2-iodophenyl)benzamide, **5c**[18]. 2-iodoaniline (5.49 g, 25.05 mmol) was treated with benzoyl chloride (2.9 mL, 24.98 mmol) according to the general procedure. Recrystalization of the crude product from hexanes/CHCl_3_ afforded a yellowish solid. Yield: 6.55 g (81%).

*N*-(2-iodophenyl)cyclohexanecarboxamide, **5d**[18]. 2-iodoaniline (5.50 g, 25.11 mmol) was treated with cyclohexanecarbonyl chloride (3.4 mL, 25.04 mmol) according to the general procedure. Recrystalization of the crude product from hexanes/CHCl_3_ afforded an off-white solid. Yield: 6.13 g (74%).

*N*-(2-iodophenyl)isobutyramide, **5e**[18]. 2-iodoaniline (5.49 g, 25.06 mmol) was treated with isobutyryl chloride (2.70 mL, 25.59 mmol) according to the general procedure. Recrystalization of the crude product from hexanes/CHCl_3_ afforded an off-white solid. Yield: 5.46 g (75%).

*N*-(2-iodophenyl)butyramide, **5f**[18]. 2-iodoaniline (2.19 g, 10.01 mmol) was treated with butyryl chloride (1.0 mL, 10.26 mmol) according to the general procedure. Recrystalization of the crude product from hexanes/CHCl_3_ afforded an off-white solid. Yield: 2.00 g (69%).

(*E*)*-N*-(2-(4-chlorostyryl)phenyl)acetamide, **6a**[22]. Compound **5a** (0.30 g, 1.15 mmol) was dissolved in 50 mL dry DMF. The solution was heated up to 120 °C and refluxed under nitrogen for a few minutes. Palladium (II) acetate (2.6 mg, 0.0115 mmol) was added, followed by triethylamine (0.48 mL, 3.45 mmol) into the mixture. Compound **3a** (0.20 g, 1.37 mmol) was added into the reaction flask. The mixture was reflux under nitrogen until consumption of compound **5a** (check via TLC). The reaction mixture was treated according to the general procedure. Purification of the crude product by column chromatography (dichloromethane/ethyl acetate, 97:3) afforded an off-white solid. Yield: 0.11 g (35%).

(*E*)*-N*-(2-(3-chlorostyryl)phenyl)acetamide, **6b**. Compound **5a** (0.30 g, 1.15 mmol) was dissolved in 50 mL dry DMF. The solution was heated up to 120 °C and refluxed under nitrogen for a few minutes. Palladium (II) acetate (2.6 mg, 0.0115 mmol) was added, followed by triethylamine (0.48 mL, 3.45 mmol) into the mixture. Compound **3b** (0.17 g, 1.20 mmol) was added into the reaction flask. The mixture was reflux under nitrogen until consumption of compound **5a** (check via TLC). The reaction mixture was treated according to the general procedure. Purification of the crude product by column chromatography (dichloromethane/ethyl acetate, 97:3) afforded an off-white solid. Yield: 0.09 g (27%).

(*E*)*-N*-(2-(4-fluorostyryl)phenyl)acetamide, **6c**. Compound **5a** (0.30 g, 1.15 mmol) was dissolved in 50 mL dry DMF. The solution was heated up to 120 °C and refluxed under nitrogen for a few minutes. Palladium (II) acetate (2.6 mg, 0.0115 mmol) was added, followed by triethylamine (0.48 mL, 3.45 mmol) into the mixture. Compound **3c** (0.15 g, 1.20 mmol) was added into the reaction flask. The mixture was reflux under nitrogen until consumption of compound **5a** (check via TLC). The reaction mixture was treated according to the general procedure. Purification of the crude product by column chromatography (dichloromethane/ethyl acetate, 97:3) afforded an off-white solid. Yield: 0.13 g (42%).

(*E*)*-N*-(2-(3-fluorostyryl)phenyl)acetamide, **6d**. Compound **5a** (1.0 g, 3.83 mmol) was dissolved in 20 mL dry DMF. The solution was heated up to 120 °C and refluxed under nitrogen for a few minutes. Palladium (II) acetate (17.2 mg, 0.0767 mmol) was added, followed by triethylamine (1.9 mL, 13.41 mmol) into the mixture. Compound **3d** (0.49 g, 4.00 mmol) was added into the reaction flask. The mixture was reflux under nitrogen until consumption of compound **5a** (check via TLC). The reaction mixture was treated according to the general procedure. Purification of the crude product by column chromatography (dichloromethane/ethyl acetate, 97:3) afforded an off-white solid. Yield: 0.20 g (20%).

(*E*)*-N*-(2-(4-bromostyryl)phenyl)acetamide, **6e**. Compound **5a** (0.30 g, 1.15 mmol) was dissolved in 80 mL dry DMF. The solution was heated up to 120 °C and refluxed under nitrogen for a few minutes. Palladium (II) acetate (2.6 mg, 0.0115 mmol) was added, followed by triethylamine (0.7 mL, 4.6 mmol) into the mixture. Compound **3e** (0.2288 g, 1.25 mmol) was added into the reaction flask. The mixture was reflux under nitrogen until consumption of compound **5a** (check via TLC). The reaction mixture was treated according to the general procedure. Purification of the crude product by column chromatography (dichloromethane/ethyl acetate, 97:3) afforded a yellow solid. Yield: 0.13 g (37%).

(*E*)*-N*-(2-(3,5-dimethoxystyryl)phenyl)acetamide, **6f**. Compound **5a** (1.02 g, 3.93 mmol) was dissolved in 10 mL dry DMF. The solution was heated up to 120 °C and refluxed under nitrogen for a few minutes. Palladium (II) acetate (16.5 mg, 0.0735 mmol) was added, followed by triethylamine (1.94 mL, 13.9 mmol) into the mixture. Compound **3f** (0.78g, 4.77 mmol) was added into the reaction flask. The mixture was reflux under nitrogen until consumption of compound **5a** (check via TLC). The reaction mixture was treated according to the general procedure. Purification of the crude product by column chromatography (hexane/ethyl acetate, 60:40) afforded an off-white solid. Yield: 0.52 g (45%).

(*E*)-*N*-(2-(2-methoxystyryl)phenyl)furan-2-carboxamide, **6g**[15]. Compound **5b** (1.51 g, 4.81 mmol) was dissolved in 25 mL dry DMF. The solution was heated up to 120 °C and refluxed under nitrogen for a few minutes. Palladium (II) acetate (11.2 mg, 0.0499 mmol) was added, followed by triethylamine (2.4 mL, 17.22 mmol) into the mixture. Compound **3g** (0.83 g, 6.16 mmol) was added into the reaction flask. The mixture was reflux under nitrogen until consumption of compound **5b** (check via TLC). The reaction mixture was treated according to the general procedure. Purification of the crude product by column chromatography (hexane/ethyl acetate, 60:40) afforded a white solid. Yield: 0.75 g (49%).

(*E*)-*N*-(2-(3-methoxystyryl)phenyl)furan-2-carboxamide, **6h**[15]. Compound **5b** (0.44 g, 1.41 mmol) was dissolved in 20 mL dry DMF. The solution was heated up to 120 °C and refluxed under nitrogen for a few minutes. Palladium (II) acetate (3.6 mg, 0.0160 mmol) was added, followed by triethylamine (0.8 mL, 5.74 mmol) into the mixture. Compound **3h** (0.30 g, 2.24 mmol) was added into the reaction flask. The mixture was reflux under nitrogen until consumption of compound **5b** (check via TLC). The reaction mixture was treated according to the general procedure. Purification of the crude product by column chromatography (hexane/ethyl acetate, 60:40) afforded a white solid. Yield: 0.15 g (33%).

(*E*)-*N*-(2-(4-methoxystyryl)phenyl)furan-2-carboxamide, **6i**[15]. Compound **5b** (0.52 g, 1.65 mmol) was dissolved in 20 mL dry DMF. The solution was heated up to 120 °C and refluxed under nitrogen for a few minutes. Palladium (II) acetate (3.6 mg, 0.0160 mmol) was added, followed by triethylamine (0.8 mL, 5.74 mmol) into the mixture. Compound **3i** (0.32 g, 2.39 mmol) was added into the reaction flask. The mixture was reflux under nitrogen until consumption of compound **5b** (check via TLC). The reaction mixture was treated according to the general procedure. Purification of the crude product by column chromatography (hexane/ethyl acetate, 60:40) afforded a white solid. Yield: 0.19 g (36%).

(*E*)-*N*-(2-(3,4-dimethoxystyryl)phenyl)furan-2-carboxamide, **6j**[15]. Compound **5b** (0.57 g, 1.83 mmol) was dissolved in 25 mL dry DMF. The solution was heated up to 120 °C and refluxed under nitrogen for a few minutes. Palladium (II) acetate (5.2 mg, 0.0232 mmol) was added, followed by triethylamine (1.2 mL, 8.61 mmol) into the mixture. Compound **3j** (0.35 g, 2.14 mmol) was added into the reaction flask. The mixture was reflux under nitrogen until consumption of compound **5b** (check via TLC). The reaction mixture was treated according to the general procedure. Purification of the crude product by column chromatography (hexane/ethyl acetate, 60:40) afforded a white solid. Yield: 0.10 g (16%).

(*E*)*-N*-(2-(3,5-dimethoxystyryl)phenyl)furan-2-carboxamide, **6k**[15]. Compound **5b** (0.57 g, 1.83 mmol) was dissolved in 25 mL dry DMF. The solution was heated up to 120 °C and refluxed under nitrogen for a few minutes. Palladium (II) acetate (5.2 mg, 0.0232 mmol) was added, followed by triethylamine (1.2 mL, 8.61 mmol) into the mixture. Compound **3f** (0.35 g, 2.14 mmol) was added into the reaction flask. The mixture was reflux under nitrogen until consumption of compound **5b** (check via TLC). The reaction mixture was treated according to the general procedure. Purification of the crude product by column chromatography (hexane/ethyl acetate, 60:40) afforded a white solid. Yield: 0.10 g (17%).

(*E*)-*N*-(2-(2-(biphenyl-4-yl)vinyl)phenyl)furan-2-carboxamide, **6l**[15]. Compound **5b** (0.37 g, 1.18 mmol) was dissolved in 20 mL dry DMF. The solution was heated up to 120 °C and refluxed under nitrogen for a few minutes. Palladium (II) acetate (3.2 mg, 0.0143 mmol) was added, followed by triethylamine (0.7 mL, 4.66 mmol) into the mixture. Compound **3k** (0.20 g, 1.21 mmol) was added into the reaction flask. The mixture was reflux under nitrogen until consumption of compound **5b** (check via TLC). The reaction mixture was treated according to the general procedure. Purification of the crude product by column chromatography (hexane/ethyl acetate, 90:10) afforded a yellowish solid. Yield: 0.04 g (9%).

(*E*)*-N-*(2-(2-([1,1′-biphenyl]-4-yl)vinyl)phenyl)benzamide, **6m**[15]. Compound **5c** (0.17 g, 0.5106 mmol) was dissolved in 20 mL dry DMF. The solution was heated up to 120 °C and refluxed under nitrogen for a few minutes. Palladium (II) acetate (2.5 mg, 0.0111 mmol) was added, followed by triethylamine (1.0 mL, 7.17 mmol) into the mixture. Compound **3k** (0.10 g, 0.5570 mmol) was added into the reaction flask. The mixture was reflux under nitrogen until consumption of compound **5c** (check via TLC). The reaction mixture was treated according to the general procedure. Purification of the crude product by column chromatography (hexane/ethyl acetate, 90:10) afforded a white solid. Yield: 0.06 g (31%).

(*E*)-*N*-(2-(3,4-dimethoxystyryl)phenyl)benzamide, **6n**[15]. Compound **5c** (0.51 g, 1.58 mmol) was dissolved in 20 mL dry DMF. The solution was heated up to 120 °C and refluxed under nitrogen for a few minutes. Palladium (II) acetate (3.5 mg, 0.0156 mmol) was added, followed by triethylamine (0.8 mL, 5.74 mmol) into the mixture. Compound **3j** (0.37 g, 2.24 mmol) was added into the reaction flask. The mixture was reflux under nitrogen until consumption of compound **5c** (check via TLC). The reaction mixture was treated according to the general procedure. Purification of the crude product by column chromatography (hexane/ethyl acetate, 80:20) afforded a white solid. Yield: 0.10 g (18%).

(*E*)*-N*-(2-(4-methoxystyryl)phenyl)benzamide, **6o**. Compound **5c** (0.51 g, 1.58 mmol) was dissolved in 20 mL dry DMF. The solution was heated up to 120 °C and refluxed under nitrogen for a few minutes. Palladium (II) acetate (3.5 mg, 0.0156 mmol) was added, followed by triethylamine (0.8 mL, 5.74 mmol) into the mixture. Compound **3i** (0.32 g, 2.39 mmol) was added into the reaction flask. The mixture was reflux under nitrogen until consumption of compound **5c** (check via TLC). The reaction mixture was treated according to the general procedure. Purification of the crude product by column chromatography (hexane/ethyl acetate, 80:20) afforded a white solid. Yield: 0.15 g (28%).

(*E*)-*N*-(2-(3,4-dimethoxystyryl)phenyl)cyclohexanecarboxamide, **6p**[15]. Compound **5d** (0.21 g, 0.63 mmol) was dissolved in 20 mL dry DMF. The solution was heated up to 120 °C and refluxed under nitrogen for a few minutes. Palladium (II) acetate (2.1 mg, 0.0094 mmol) was added, followed by triethylamine (0.5 mL, 3.59 mmol) into the mixture. Compound **3j** (0.15 g, 0.93 mmol) was added into the reaction flask. The mixture was reflux under nitrogen until consumption of compound **5d** (check via TLC). The reaction mixture was treated according to the general procedure. Purification of the crude product by column chromatography (hexane/ethyl acetate, 80:20) afforded a white solid. Yield: 0.07 g (31%).

(*E*)-*N*-(2-(3,4-dimethoxystyryl)phenyl)isobutyramide, **6q**[15]. Compound **5e** (1.49 g, 5.16 mmol) was dissolved in 25 mL dry DMF. The solution was heated up to 120 °C and refluxed under nitrogen for a few minutes. Palladium (II) acetate (11.9 mg, 0.053 mmol) was added, followed by triethylamine (2.8 mL, 20.09 mmol) into the mixture. Compound **3j** (1.02 g, 6.23 mmol) was added into the reaction flask. The mixture was reflux under nitrogen until consumption of compound **5e** (check via TLC). The reaction mixture was treated according to the general procedure. Purification of the crude product by column chromatography (hexane/ethyl acetate, 80:20) afforded an off-white solid. Yield: 0.4505 g (27%).

(*E*)*-N*-(2-(4-methoxystyryl)phenyl)isobutyramide, **6r**. Compound **5e** (0.31 g, 1.09 mmol) was dissolved in 20 mL dry DMF. The solution was heated up to 120 °C and refluxed under nitrogen for a few minutes. Palladium (II) acetate (2.4 mg, 0.0011 mmol) was added, followed by triethylamine (0.6 mL, 4.30 mmol) into the mixture. Compound **3i** (0.22 g, 1.37 mmol) was added into the reaction flask. The mixture was reflux under nitrogen until consumption of compound **5e** (check via TLC). The reaction mixture was treated according to the general procedure. Purification of the crude product by column chromatography (hexane/ethyl acetate, 80:20) afforded an off-white solid. Yield: 0.05 g (14%).

(*E*)-*N*-(2-(3,4-dimethoxystyryl)phenyl)butyramide, **6s**[15]. Compound **5f** (0.31 g, 1.09 mmol) was dissolved in 20 mL dry DMF. The solution was heated up to 120 °C and refluxed under nitrogen for a few minutes. Palladium (II) acetate (2.4 mg, 0.0011 mmol) was added, followed by triethylamine (0.6 mL, 4.30 mmol) into the mixture. Compound **3j** (0.22 g, 1.37 mmol) was added into the reaction flask. The mixture was reflux under nitrogen until consumption of compound **5f** (check via TLC). The reaction mixture was treated according to the general procedure. Purification of the crude product by column chromatography (hexane/ethyl acetate, 80:20) afforded an off-white solid. Yield: 0.05 g (15%).
